# Multi-Platform Multivariate Regression with Group Sparsity for High-Dimensional Data Integration

**DOI:** 10.3390/e28020135

**Published:** 2026-01-23

**Authors:** Shanshan Qin, Guanlin Zhang, Xin Gao, Yuehua Wu

**Affiliations:** 1 School of Statistics, Tianjin University of Finance and Economics, Tianjin 300222, China; qinss@tjufe.edu.cn; 2 Department of Mathematics and Statistics, York University, Toronto, ON M3J 1P3, Canadaxingao@yorku.ca (X.G.)

**Keywords:** multi-platform, multivariate regression, group sparsity, high-dimensional, data integration

## Abstract

High-dimensional regression with multivariate responses poses significant challenges when data are collected across multiple platforms, each with potentially correlated outcomes. In this paper, we introduce a multi-platform multivariate high-dimensional linear regression (MM-HLR) model for simultaneously modeling within-platform correlation and cross-platform information fusion. Our approach incorporates a mixture of Lasso and group Lasso penalties to promote both individual predictor sparsity and cross-platform group sparsity, thereby enhancing interpretability and estimation stability. We develop an efficient computational algorithm based on iteratively reweighted least squares and block coordinate descent to solve the resulting regularized optimization problem. We establish theoretical guarantees for our estimator, including oracle bounds on prediction error, estimation accuracy, and support recovery under mild conditions. Our simulation studies confirm the method’s strong empirical performance, demonstrating low bias, small variance, and robustness across various dimensions. The analysis of real financial data further validates the performance gains achieved by incorporating multivariate responses and integrating data across multiple platforms.

## 1. Introduction

As data complexity continues to expand in the big-data era, modern datasets increasingly display heterogeneity, high dimensionality, and multiple sources. A common example arises when experiments with the same scientific objective are conducted across different platforms or environments [1,2,3,4]. Modeling each dataset individually may result in failing to capture their intrinsic connections, which stem from their shared goal. This motivates the development of methods capable of integrating multi-platform data for unified, simultaneous analysis.

Data integration provides a timely solution by leveraging multiple data sources to enable more robust and efficient statistical inference than relying on any single source alone [5]. In a systematic review, Ref. [5] surveyed integration methods for combining probability samples with non-probability samples and big data sources (see also the references therein). Within the regression framework, a series of studies have advanced data integration methodologies. For instance, Ref. [1] introduced a pseudolikelihood information criterion for high-dimensional multi-experiment data with mixed response types and varying predictor measurements, establishing selection consistency even with unbounded model size and demonstrating through simulations that data integration substantially outperforms single-source analysis. Building on this work, Ref. [6] implemented the FusionLearn R package, which provides a fusion learning algorithm for cross-platform data analysis. Ref. [7] extended the framework to multi-platform data with sub-Gaussian or sub-exponential errors, developing a consistent model selection criterion based on composite likelihood and Bayesian posterior probabilities to recover the union support of predictors under diverging model dimensions. Meanwhile, Ref. [4] addressed multi-task feature learning with mixed continuous and discrete responses using a mixed $\ell_{2,1}$-regularized composite quasi-likelihood function. In a related vein, Ref. [8] proposed a quantile regression approach for high-dimensional multi-source data exhibiting heterogeneity and heavy-tailed error distributions, providing both theoretical guarantees and practical advantages in model recovery.

However, much of the existing literature focuses primarily on univariate response modeling within a single laboratory or experiment. In many modern applications, response variables are multivariate and correlated, yet share the same set of high-dimensional covariates or predictors [9]. For instance, in the UK Biobank population-based cohort study, researchers face large-scale, ultrahigh-dimensional features alongside a wide array of correlated phenotypic outcomes, including lifestyle measures, biomarkers, and disease diagnoses [10]. Such data structures have motivated extensive methodological developments in high-dimensional multivariate regression. Seminal work includes remMap by [11], designed for multivariate response regression in high-dimension–low-sample-size settings. Ref. [9] proposed a blockwise descent algorithm for group-penalized multi-response regression. Further advancing the field, Ref. [12] introduced a regularization method that enhances variable selection by efficiently eliminating irrelevant blocks of regression coefficients. More recently, Ref. [10] developed a scalable sparse reduced-rank regression method for high-dimensional multi-task learning with correlated outcomes. Ref. [13] proposed a novel framework suited for settings with large numbers of responses, response categories, and predictors. Additionally, several quantile regression approaches have been proposed to handle multiple responses under non-Gaussian or heterogeneous error settings, like [14,15], among others.

Nevertheless, relatively few works have simultaneously addressed the complexities of multivariate responses across multiple data sources. We thus propose a multi-platform multivariate high-dimensional linear regression (MM-HLR) method, designed to jointly model within-platform correlation while promoting cross-platform group sparsity. Structured or group sparsity has been well studied recently. Zhang et al. [16] presented a probabilistic framework for subset selection under partition constraints, which aligns with the group-sparsity and cross-platform fusion objectives of our study. In addition, Li et al. [12] proposed a penalized multivariate multiple linear regression model with an arbitrary group structure for the regression coefficient matrix. Besides, the mixed $\ell_{2,1}$ penalty has similarly been employed for multitask feature learning and selection [4,17]. Kawano et al. [18] proposed a multivariate regression modeling in integrative analysis that performed group selection by group lasso estimation. In this article, we extended the mixture of $\ell_{1}$ and $\ell_{2}$ penalty formulation from [12] to simultaneously enforce individual sparsity and cross-platform group sparsity in our multi-platform setting. An efficient optimization framework, combining block coordinate descent with a proxy approximation strategy, is introduced to solve the resulting non-convex regularized problem. Theoretically, we establish non-asymptotic bounds on the prediction error, estimation accuracy, and support recovery of the MM-HLR estimator. Empirically, we evaluate the proposed method under varying sample sizes and predictor dimensionalities, benchmarking it against two alternative approaches: (i) FusionLearn [6], which is designed for multi-platform integration but treats responses as univariate, thereby ignoring within-platform correlation structures, and (ii) multivariate Group Lasso [19], which is designed for multivariate regression within a single platform and thus overlooks cross-platform grouping. Comprehensive simulation studies are conducted to demonstrate the method’s performance across diverse data-generating scenarios. Finally, we validate the proposed method through a real-world financial data analysis to assess the gains of jointly modeling multivariate responses and integrating data across multiple platforms.

The remainder of this article is organized as follows: Section 2 introduces the model framework and parameter estimation. Section 3 establishes the theoretical guarantees of the proposed estimator. Section 4 presents simulation studies to evaluate the performance of the method. We provide a real data analysis in Section 5. Finally, Section 6 concludes this article.

## 2. Methodology

Let A and B be two matrices. A⪯B denotes that B−A is positive semidefinite. ${∥A∥}_{F}$ denotes the Frobenius norm of A. In addition, ${∥A∥}_{1}$ and ${\left||A|\right|}_{\infty }$ denote the sum and the maximum of the absolute values of all entries of A. Let a be a vector. We denote its $\ell_{2}$ and $\ell_{1}$ norms by ${\left||a|\right|}_{2}$ and ${\left||a|\right|}_{1}$, respectively.

### 2.1. Model Setup

Assume we have *n* objects and aim to model the linear relationship between *m* responses and *p* predictors for each object. To enhance measurement accuracy, each object is sent to *k* different platforms, each of which returns two matrices, a response matrix $Y_{i}\in R^{n\times m}$ and a design matrix $X_{i}\in R^{n\times p}$, i=1,…,k. Due to variations in equipment and measurement protocols across platforms, the scale and continuity of the results may differ [1,6]. Our goal is to identify the common set of influential predictors that affect the responses across all platforms. Moreover, because multivariate responses are measured on the same objects, there exist unknown correlations between responses within the platform and across platforms. Key terminology is summarized in Table 1.

From the linearity relationship between responses and predictors, we have the following:

(1) $$\begin{array}{c} Y_{i}=X_{i}B_{i}+E_{i},i=1,\ldots ,k \end{array}$$

where $Y_{i}\in R^{n\times m}$, $X_{i}\in R^{n\times p}$, $B_{i}\in R^{p\times m}$ are respectively the response matrix, design matrix and regression parameter matrix from *i*th platform. $E_{i}\in R^{n\times m}$ is the error matrix, where the rows of $E_{i}$ are identically independent from $N_{m}\left(0,\Sigma_{i}\right)$, with $\Sigma_{i}\in R^{m\times m}$ being the within-platform error covariance matrix.

To simplify the modeling complexity, we treat each platform independently within the likelihood function. Accordingly, we adopt a marginal composite likelihood approach to integrate information across platforms [7,20,21], i.e., the following is calculated:

(2) $$\begin{array}{c} L\left(B\right)=\prod_{i=1}^{k}f\left(Y_{i};B_{i}\right), \end{array}$$

where $B=\left(B_{1},\ldots ,B_{k}\right)\in R^{p\times km}$, and $f(Y_{i};B_{i})$ denotes the multivariate normal density function of $Y_{i}$, that is,

(3) $$\begin{array}{c} f\left(Y_{i};B_{i}\right)\propto |\Sigma_{i}{|}^{-\frac{n}{2}}\exp\left\{-\frac{1}{2}\mathrm{tr}\left[\left(Y_{i}-X_{i}B_{i}\right)\Sigma_{i}^{-1}{\left(Y_{i}-X_{i}B_{i}\right)}^{⊤}\right]\right\}. \end{array}$$

The log-likelihood function can be given by the following (excluding the constant):

(4) $$\begin{array}{c} \ell \left(B\right)=-\frac{n}{2}\sum_{i=1}^{k}\log\left|\Sigma_{i}\right|-\frac{1}{2}\sum_{i=1}^{k}\mathrm{tr}\left[\left(Y_{i}-X_{i}B_{i}\right)\Sigma_{i}^{-1}{\left(Y_{i}-X_{i}B_{i}\right)}^{⊤}\right]. \end{array}$$

Following [1], the objective of this study is to recover a union subset of predictors, where each selected predictor is associated with at least one outcome across the platforms. To identify these commonly influential predictors, we enforce the constraint that the set of nonzero rows of $B_{k}$ is identical for all platforms k=1,…,K. Stacking the *r*-th row vector of all $B_{i}$s, we obtain the regression coefficient matrix for predictor *r* across all *m* responses and *k* platforms, i.e., written as follows:

$$\Theta^{\left(r\right)}={\left(\theta_{1}^{\left(r\right)},\ldots ,\theta_{k}^{\left(r\right)}\right)}^{⊤}\in R^{k\times m},$$

where $\theta_{i}^{\left(r\right)}\in R^{m\times 1}$ represents the *r*-th row of $B_{i}$. We assume that the sparsity among *k* platforms is the same with respect to the predictors, that is, $\theta_{1}^{\left(r\right)},\ldots ,\theta_{k}^{\left(r\right)}$ are either all zero or all nonzero for each r=1,…,p. To select the influential predictors among all platforms, we add the group lasso penalty into the log-likelihood function, i.e., written as follows:

(5) $$\begin{array}{c} \tilde{Q}\left(B\right)=\ell \left(B\right)-n\sum_{j=1}^{p}\Omega_{\lambda_{n}}\left(|\right|\Theta^{\left(r\right)}{\left|\right|}_{F}), \end{array}$$

where $\left|\right|\cdot {\left|\right|}_{F}$ is the Frobenius norm (square root of sum of squares of all entries). Assuming the penalty function is the mixture of $L_{1}$ and $L_{2}$ norms, then a sparse estimate of B (denoted as $\hat{B}$ ) can be obtained by solving the minimization problem as follows:

(6) $$\begin{array}{c} \min_{B}\tilde{Q}\left(B\right)=\min_{B}-\;\ell \left(B\right)+n\sum_{i,r,s}\lambda_{irs}|\theta_{irs}|+n\sum_{r=1}^{p}\lambda_{g}\left|\right|\Theta^{\left(r\right)}{\left|\right|}_{F}. \end{array}$$

Denote $p_{1}\left(B\right)=n\sum_{i,r,s}\lambda_{irs}\left|\theta_{irs}\right|$ and $p_{2}\left(B\right)=n\sum_{r=1}^{p}\lambda_{g}\left|\right|\Theta^{\left(r\right)}{\left|\right|}_{F}$. The first penalty $p_{1}\left(B\right)$ encourages sparsity (individual coefficients to zero), and the second penalty $p_{2}\left(B\right)$ encourages group sparsity for each predictor to be zero across all platforms.

### 2.2. Parameter Estimation

We use an iteratively reweighted least squares approach and block coordinate descent per predictor r, r=1,…,p. For simplicity, we assume that the element-wise regularization parameters for the Lasso penalty are identical, $\lambda_{irs}=\lambda_{e}$. In practice, the covariance matrix $\Sigma_{i}$ among responses is typically unknown for each platform i=1,…,k. A simple initial estimate, such as the empirical covariance of the model residuals, can be employed. The following algorithm is then implemented conditional on these estimated $\Sigma_{i}$s. Denote $f\left(B_{i}\right)=\frac{1}{2}\mathrm{tr}\left(\left(Y_{i}-X_{i}B_{i}\right)\Sigma_{i}^{-1}{\left(Y_{i}-X_{i}B_{i}\right)}^{⊤}\right)$. Then the optimization problem (6) becomes the following:

(7) $$\begin{array}{c} \min_{B}Q\left(B\right)=\min_{B}\sum_{i=1}^{k}f\left(B_{i}\right)+n\sum_{i,r,s}\lambda_{e}|\theta_{irs}|+n\sum_{r=1}^{p}\lambda_{g}\left|\right|\Theta^{\left(r\right)}{\left|\right|}_{F}. \end{array}$$

Rewrite $X_{i}B_{i}=\sum_{r=1}^{p}X_{i}^{\left(r\right)}\theta_{i}^{(r)⊤}$. For predictor *r*, by holding $\Theta^{\left(j\right)}$ fixed for j≠r, we define residuals without predictor *r* for platform *i*, $R_{i}^{\left(r\right)}=Y_{i}-\sum_{j\neq r}X_{i}^{\left(j\right)}\theta_{i}^{(j)⊤}\in R^{n\times m}$. Then the first gradient of *f*, with respect to $\theta_{i}^{\left(r\right)}$, is $\nabla_{\theta_{i}^{\left(r\right)}}f=-\Sigma_{i}^{-1}R_{i}^{(r)⊤}X_{i}^{\left(r\right)}\in R^{m\times 1}$, where $X_{i}^{\left(j\right)}\in R^{n\times 1}$ is the column *j* of $X_{i},\;i=1,\ldots ,k$. Denote the following:

(8) $$\begin{array}{c} S_{i}^{\left(r\right)}=-\nabla_{\theta_{i}^{\left(r\right)}}f=\Sigma_{i}^{-1}R_{i}^{(r)⊤}X_{i}^{\left(r\right)}\in R^{m\times 1}. \end{array}$$

By coordinate descent algorithm, we estimate $\theta_{i}^{\left(r\right)}$ by fixing the rest. Let $\Theta^{\left(j\right)}$ be fixed for j≠r, then problem (7) can be transformed to be a minimization sub-problem for predictor r(r=1,…,p) across *k* platforms, i.e., the following is calculated:

(9) $$\min_{\theta_{i}^{\left(r\right)}}Q_{r}\left(\theta_{i}^{\left(r\right)}\right)=\sum_{i=1}^{k}\left(\frac{∥X_{i}^{\left(r\right)}{∥}_{2}^{2}}{2}\;\theta_{i}^{(r)⊤}\Sigma_{i}^{-1}\theta_{i}^{\left(r\right)}-S_{i}^{(r)⊤}\theta_{i}^{\left(r\right)}+\lambda_{e}n{∥\theta_{i}^{\left(r\right)}∥}_{1}\right)+\lambda_{g}n{∥\Theta^{\left(r\right)}∥}_{F}.$$

Next, we will show how to solve the sub-problem (9). Define the element-wise soft-thresholding $S_{i,soft}^{\left(r\right)}=S_{n\lambda_{e}}\left(S_{i}^{\left(r\right)}\right)$, where $S_{\tau }\left(x\right)=\mathrm{sign}\left(x\right)\max\left(|x|-\tau ,0\right)$, a soft-thresholding operator. In light of the block soft-thresholding solution of the group Lasso in [22], the predictor *r* is set to be inactive, $\theta_{i}^{\left(r\right)}=0$ across all platforms if the following is met:

(10) $$\begin{array}{c} T_{r}=\sum_{i=1}^{k}{∥S_{i,soft}^{\left(r\right)}∥}_{2}^{2}\leq n^{2}\lambda_{g}^{2}. \end{array}$$

Otherwise, the predictor *r* is selected to be active, $∥\Theta^{\left(r\right)}{∥}_{F}\neq 0$. If so, we can update $\theta_{i}^{\left(r\right)}$ via a block soft-thresholding operator, as shown in [23]. Given our objective to select a union subset of active predictors across all platforms, an active predictor *r* may be relevant to platform *i* or other platforms. We therefore consider the following two scenarios for updating $\theta_{i}^{\left(r\right)}$.

Assume that the predictor *r* in all platforms except platform *i* is inactive, that is, $\theta_{i^{\prime }}^{\left(r\right)}=0,$ for $i^{\prime }\neq i.$ Then the group Lasso norm reduces to the following: $∥\Theta^{\left(r\right)}{∥}_{F}={∥\theta_{i}^{\left(r\right)}∥}_{2},$ and the sub-problem (9) simplifies to become

(11) $$\begin{array}{c} \min_{\theta_{i}^{\left(r\right)}\in R^{m}}\frac{∥X_{i}^{\left(r\right)}{∥}_{2}^{2}}{2}\;\theta_{i}^{(r)⊤}\;\Sigma_{i}^{-1}\;\theta_{i}^{\left(r\right)}-S_{i}^{(r)⊤}\theta_{i}^{\left(r\right)}+n\lambda_{e}∥\theta_{i}^{\left(r\right)}{∥}_{1}+n\lambda_{g}{∥\theta_{i}^{\left(r\right)}∥}_{2}, \end{array}$$

where the objective function is a sum of a smooth quadratic term and a composite nonsmooth penalty $\lambda_{e}{∥\cdot ∥}_{1}+\lambda_{g}{∥\cdot ∥}_{2}$. Its proximal mapping consists of sequential soft-thresholding followed by block shrinkage. Then the minimizer of the row-wise objective with respect to $\theta_{i}^{\left(r\right)}$ is given by the following:

(12) $$\theta_{i}^{\left(r\right)}=\Sigma_{i}\frac{S_{n(\lambda_{e}+\lambda_{g})}\left(S_{i}^{\left(r\right)}\right)}{∥X_{i}^{\left(r\right)}{∥}_{2}^{2}}.$$

Assume that the predictor *r* is active across all platforms, we conduct the closed-form updating for the nonzero group *r* as follows. A solution to problem (9) is a minimizer of the following:

(13) $${\tilde{Q}}_{r}\left(\theta_{i}^{\left(r\right)}\right)=\sum_{i=1}^{k}\left(\frac{∥X_{i}^{\left(r\right)}{∥}_{2}^{2}}{2}\;\theta_{i}^{(r)⊤}\Sigma_{i}^{-1}\theta_{i}^{\left(r\right)}-S_{i,soft}^{(r)⊤}\theta_{i}^{\left(r\right)}\right)+\lambda_{g}n{∥\Theta^{\left(r\right)}∥}_{F}.$$

Take the first derivative of ${\tilde{Q}}_{r}$ with respect to $\theta_{i}^{\left(r\right)}$ and let them be zero, i.e., as follows:

(14) $$∥X_{i}^{\left(r\right)}{∥}_{2}^{2}\;\Sigma_{i}^{-1}\theta_{i}^{\left(r\right)}-S_{i,soft}^{\left(r\right)}+\lambda_{g}n\frac{\theta_{i}^{\left(r\right)}}{∥\Theta^{\left(r\right)}{∥}_{F}}=0.$$

We rewrite the above equation to group the terms linear in $\theta_{i}^{\left(r\right)}$, written as follows:

(15) $$\left(∥X_{i}^{\left(r\right)}{∥}_{2}^{2}\;\Sigma_{i}^{-1}+\frac{\lambda_{g}n}{∥\Theta^{\left(r\right)}{∥}_{F}}I_{m}\right)\theta_{i}^{\left(r\right)}=S_{i,soft}^{\left(r\right)}.$$

which yields a closed-form of $\theta_{i}^{\left(r\right)}$, i.e.,

(16) $$\theta_{i}^{\left(r\right)}={\left(∥X_{i}^{\left(r\right)}{∥}_{2}^{2}\;\Sigma_{i}^{-1}+\frac{\lambda_{g}n}{∥\Theta^{\left(r\right)}{∥}_{F}}I_{m}\right)}^{-1}S_{i,soft}^{\left(r\right)}.$$

### 2.3. Algorithm

We estimate ${\left\{B_{i}\right\}}_{i=1}^{k}$ via a nested alternating minimization procedure that consists of an outer loop updating covariance matrices and an inner loop performing row-wise block coordinate descent on regression coefficients with sparse group penalties. For the (t+1)-th outer loop, $t=0,\ldots ,T_{\max}-1$, we perform a fixed number of inner loops to update the regression coefficients $B_{i}^{\left(t,\;\ell \right)},\ell =0,1,\ldots ,\ell_{\max}$ while keeping the covariance matrices $\Sigma_{i}^{\left(t\right)}$ fixed. When the inner loop arrives at the maximum iteration, we update $B_{i}^{\left(t+1\right)}$, followed by the updating of $\Sigma_{i}^{\left(t+1\right)}$ and recording the residuals. Convergence of the algorithm is then assessed at the outer-iteration level based on changes in $B_{i}^{\left(t+1\right)}$ and $\Sigma_{i}^{\left(t+1\right)}$. Each outer iteration, therefore, consists of a full inner loop for coefficient updates, followed by updating the regression covariance and convergence evaluation. The details are as follows:

Step 1: Initialization $B_{i}^{\left(0\right)}$ and $\Sigma_{i}^{\left(0\right)}$ for each platform i, i=1,…,k.
Step 2: (Outer loop) Update $B_{i}^{\left(t+1\right)}$ across all platforms given $\Sigma_{i}^{\left(t\right)},B_{i}^{\left(t\right)}$ at *t*-th iteration.
  Substep 2.1: (Inner loop) Initialization: for predictor r(r=1,…,p), $\theta_{i}^{\left(r)(t,0\right)}=\theta_{i}^{\left(r)(t\right)}$, i=1,…,k, where $\theta_{i}^{\left(r)(t\right)}$ is the *r*-th row of $B_{i}^{\left(t\right)}$.
  Substep 2.2: Update $\theta_{i}^{\left(r)(t,\ell +1\right)}$ across all platforms given $\theta_{i}^{\left(r)(t,\ell \right)}$ and $\Sigma_{i}^{\left(t\right)}$. Perform row-wise group screening. Let $\theta_{i}^{\left(r)(t,\ell +1\right)}=0$ if $T_{r}^{\left(t,\ell \right)}\leq n^{2}\lambda_{g}^{2}$. Otherwise, predictor *r* is added to the active row set. For each active predictor *r*, we conduct row-wise coefficient updating, i.e., calculated as follows:
  (17) $$\theta_{i}^{\left(r)(t,\ell +1/2\right)}=\Sigma_{i}^{\left(t\right)}\frac{S_{n(\lambda_{e}+\lambda_{g})}\left(S_{i}^{\left(r)(t,\ell \right)}\right)}{∥X_{i}^{\left(r\right)}{∥}_{2}^{2}}$$
  if $∥\theta_{i^{\prime }}^{\left(r)(t,\ell \right)}{∥}_{1}=0,i^{\prime }\neq i$. If not, then the following is calculated:
  (18) $$\theta_{i}^{\left(r)(t,\ell +1/2\right)}={\left(∥X_{i}^{\left(r\right)}{∥}_{2}^{2}\;\Sigma_{i}^{-1(t)}+\frac{\lambda_{g}n}{∥\Theta^{\left(r)(t,\ell \right)}{∥}_{F}}I_{m}\right)}^{-1}S_{i,soft}^{\left(r)(t,\ell \right)}.$$
  Substep 2.3: For each platform *i*, $B_{i}^{\left(t,\ell +1\right)}=\left({\hat{B}}_{i,\hat{A}},0_{{\hat{A}}^{c}}\right)$, where
  $\hat{A}=\{r:∥\theta_{i}^{\left(r)(t,\ell +1/2\right)}{∥}_{1}\neq 0\}$, and the following:
  (19) $${\hat{B}}_{i,\hat{A}}={\left(X_{i,\hat{A}}^{⊤}X_{i,\hat{A}}+\varepsilon I_{|\hat{A}|}\right)}^{-1}X_{i,\hat{A}}^{⊤}Y_{i}.$$
  Substep 2.4: Repeat substeps 2.2–2.3 until it reaches the maximum inner iterations $\ell_{\max}$.
Step 3: Update $\Sigma_{i}^{\left(t+1\right)}$ via $\Sigma_{i}^{\left(t+1\right)}=\frac{1}{n}{\left(Y_{i}-X_{i}B_{i}^{\left(t+1\right)}\right)}^{⊤}\left(Y_{i}-X_{i}B_{i}^{\left(t+1\right)}\right).$
Step 4: Repeat steps 2–3 until it meets the termination conditions, i.e., the following:
  (20) $$R_{B}^{\left(t+1\right)}=\frac{1}{k}\sum_{i=1}^{k}{∥B_{i}^{\left(t+1\right)}-B_{i}^{\left(t\right)}∥}_{F}^{2}\leq \delta ,$$
  (21) $$R_{\Sigma }^{\left(t+1\right)}=\frac{1}{k}\sum_{i=1}^{k}{∥\Sigma_{i}^{\left(t+1\right)}-\Sigma_{i}^{\left(t\right)}∥}_{F}^{2}\leq \delta ,$$

where δ is a small number, say, 1 × 10^−4^.

We remark that, for initialization, we set $B_{i}^{\left(0\right)}=0.1_{p\times m}$, $\Sigma_{i}^{\left(0\right)}=I_{m}$, for i=1,…,k. Tuning parameters are typically selected by evaluating a candidate set of values via cross-validation or an information criterion like the adjusted BIC. To enhance computational efficiency and avoid a costly two-dimensional grid search, we adopt a practical simplification by fixing the relationship between $\lambda_{g}$ and $\lambda_{e}$, for instance, by setting $\lambda_{g}=\lambda_{e}$ or $\lambda_{g}=\lambda_{e}/2$. Consequently, we select the optimal $\lambda_{e}$ via two-fold cross-validation from a common empirical candidate set for $\lambda_{e}$, such as {0.1, 0.2, ..., 2.0 } with an increment of 0.1 or smaller. The range and granularity of the candidate set can be adapted to the specific application.

## 3. Theoretical Property

The theoretical property is in light of [12], where their theories are built under the framework of multivariate linear regression. Herein, we extend them to multiple platforms. For platform i(i=1,…,k), denote $J_{1}\left(B_{i}\right)=\left\{rs:\right|\theta_{irs}\left|\neq 0\right\}$ the index set of nonzero elements in $B_{i}$, and $J_{2}\left(B\right)=\left\{r:|\right|\Theta^{\left(r\right)}{\left|\right|}_{2}\neq 0,r=1,\ldots ,p\}$ the index set of nonzero rows in B. We assume that the sparsity of all $B_{i},\;i=1,\ldots ,k$ is the same. Define $M_{1}\left(B\right)=\left|J_{1}\left(B_{i}\right)\right|$ and $M_{2}\left(B\right)=\left|J_{2}\left(B\right)\right|$. For any matrix $\Delta \in R^{p\times m}$ and $J_{1}\subseteq \left\{rs:1\leq r\leq p,1\leq s\leq m\right\}$, denote $\Delta_{J_{1}}$ as the projection of Δ on the index set $J_{1}$, which is a matrix with the same elements of Δ on coordinates $J_{1}$ and zeros on the complementary coordinates $J_{1}^{c}$. Denote $\Delta_{J_{2}}=\left\{\Theta_{\Delta }^{\left(r\right)},r\in J_{2}\right\}$, where $\Theta_{\Delta }^{\left(r\right)}$ is a p×m matrix with the *r*th row same as Δ and zeros on the other rows.

Let $B_{i}^{*}$ be the true regression coefficient matrices in model (1), and $\hat{B_{i}}$ be its estimated counterpart for i=1,…,k. Assume each column of the random error matrix follows a multivariate normal distribution $N_{m}\left(0,\Sigma_{i}^{*}\right)$. Denote $u=M_{1}\left(B^{*}\right)$ and $v=M_{2}\left(B^{*}\right)$. The theoretical framework is built on a given covariance matrix for each platform *i*. In practice, $\Sigma_{i}^{*}$ is usually unknown. So we instead use an estimator ${\hat{\Sigma }}_{i}$. Before proceeding, we impose mild conditions on both design matrices $X_{i}$ and covariance matrices $\Sigma_{i}^{*}$ for all platforms.

**Assumption** **1.** *Assume that the columns of $X_{i}$ are centered such that the diagonal elements of the matrix $X_{i}^{⊤}X_{i}/n$ are equal to 1 for all i=1,…,k. Let $\psi_{\max}>0$ be the largest eigenvalue of $X_{i}^{⊤}X_{i}/n$ for all i=1,…,k.*

**Assumption** **2.** For a given $\Sigma_{i}\in R^{m\times m}$, assume that there exists a $\lambda^{*}\in \left(0,1\right)$, such that $-\lambda^{*}I⪯\Sigma_{i}-\Sigma_{i}^{*}⪯\lambda^{*}I$ for all i=1,…,k. There exist positive constants $0<C_{1}<C_{2}$ such that $C_{1}\geq \lambda^{*}$, and the eigenvalues of $\Sigma_{i}^{*}$ are less than $C_{2}$ and larger than $C_{1}$ for all i=1,…,k.

**Assumption** **3.** *Let $J_{1}\subseteq \left\{rs:1\leq r\leq p,1\leq s\leq m\right\}$ and $J_{2}\subseteq \left\{1,\ldots ,p\right\}$ be any index sets that satisfy $|J_{1}\left|\leq u,\right|J_{2}|\leq v$. Let $\tilde{\rho }=\left\{\rho_{r},r=1,\ldots ,p\right\}$ be a set of positive numbers. For any nontrivial matrices $\Delta_{i}\in R^{p\times m},\;i=1,\ldots ,k$, if it satisfies the following:*

(22) $$\begin{array}{c} \sum_{i=1}^{k}|\Delta_{i,J_{1}^{c}}{|}_{1}+2\sum_{r\in J_{2}^{c}}\rho_{r}\left|\right|\Delta^{\left(r\right)}{\left|\right|}_{2}\leq 3\sum_{i=1}^{k}|\Delta_{i,J_{1}}{|}_{1}+2\sum_{r\in J_{2}}\rho_{r}\left|\right|\Delta^{\left(r\right)}{\left|\right|}_{2}, \end{array}$$

*where $\Delta^{\left(r\right)}$ is built by the rth rows of $\Delta_{i}$ for all i=1,…,k, then the following minimums exist and are positive, i.e., the following:*

(23) $$\begin{array}{cc} \; & \kappa_{1}\left(u,v,\tilde{\rho }\right)=\min_{J_{1},J_{2},\Delta \neq 0}\frac{\sum_{i=1}^{k}{\left(\mathrm{tr}[X_{i}\Delta_{i}\Sigma_{i}^{-1}{\left(X_{i}\Delta_{i}\right)}^{⊤}]\right)}^{1/2}}{n^{1/2}\sum_{i=1}^{k}\left|\right|\Delta_{i,J_{1}}{\left|\right|}_{F}}>0, \end{array}$$

(24) $$\begin{array}{cc} \; & \kappa_{2}\left(u,v,\tilde{\rho }\right)=\min_{J_{1},J_{2},\Delta \neq 0}\frac{\sum_{i=1}^{k}{\left(\mathrm{tr}[X_{i}\Delta_{i}\Sigma_{i}^{-1}{\left(X_{i}\Delta_{i}\right)}^{⊤}]\right)}^{1/2}}{n^{1/2}\left|\right|\Delta_{J_{2}}{\left|\right|}_{F}}>0. \end{array}$$

We remark that these constants involved in assumptions represent standard regularity conditions in statistical theory. While their specific values are derived from the proofs and are not tuned in practice, their existence is required to establish the desired theoretical guarantees. Furthermore, we define $s_{{\tilde{\rho }}^{*}}=\sum_{r\in J_{2}\left(B^{*}\right)}\rho_{r}^{2}$, $\sigma^{2}=\frac{C_{1}}{{\left(C_{1}-\lambda^{*}\right)}^{2}},\;\lambda_{g}=\rho_{r}\lambda$ for $r=1,\ldots ,p,\;\rho =\min_{r}\left\{1,\rho_{r}\right\}$, and $\lambda =2\sigma A{\left\{\log\left(pmk\right)/n\right\}}^{1/2}$ for some constant $A>\sqrt{2}$. Rewrite $\kappa_{1}=\kappa_{1}\left(u,v,\tilde{\rho }\right)$ and $\kappa_{2}=\kappa_{2}\left(u,v,\tilde{\rho }\right)$.

**Theorem** **1.** *Under Assumptions 1–3, the following oracle bounds for the prediction error, the estimation error, and the order of sparsity hold, with probability at least $1-{\left(pmk\right)}^{1-A^{2}/2}$, i.e., written as follows:*

(25) $$\begin{array}{cc} & \frac{1}{n}\sum_{i=1}^{k}\mathrm{tr}\left[\left(X_{i}B_{i}^{*}-X_{i}{\hat{B}}_{i}\right)\Sigma_{i}^{-1}{\left(X_{i}B_{i}^{*}-X_{i}{\hat{B}}_{i}\right)}^{⊤}\right]\leq 16\lambda^{2}k{\left(\frac{u^{1/2}}{\kappa_{1}}+\frac{s_{{\tilde{\rho }}^{*}}^{1/2}}{\kappa_{2}}\right)}^{2}, \end{array}$$

(26) $$\begin{array}{cc} \sum_{i=1}^{k}\left|\right|{\hat{B}}_{i}-B_{i}^{*}{\left|\right|}_{1}\leq \frac{24\lambda k^{1/2}}{1+\rho }{\left(\frac{u^{1/2}}{\kappa_{1}}+\frac{s_{{\tilde{\rho }}^{*}}^{1/2}}{\kappa_{2}}\right)}^{2}, & \end{array}$$

(27) $$\begin{array}{cc} M_{1}\left({\hat{B}}_{i}\right)\leq \frac{64\psi_{\max}}{\left(C_{1}-\lambda^{*}\right)}{\left(\frac{u^{1/2}}{\kappa_{1}}+\frac{s_{{\tilde{\rho }}^{*}}^{1/2}}{\kappa_{2}}\right)}^{2},i=1,\ldots ,k. & \end{array}$$

The proof of Theorem 1 is given in the Appendix A. The three inequalities (25)–(27) guarantee the performance of the estimator $\hat{B}$ in terms of prediction accuracy, estimation error, and sparsity. The first inequality (25) bounds the weighted mean squared prediction error across all *k* platforms. The bound scales with log(pmk)/n and depends on the sparsity and grouping structures. The second inequality (26) controls the cumulative $\ell_{1}$-norm of ${\hat{B}}_{i}$ from $B_{i}^{*}$ across all platforms. This bound grows slowly with $\sqrt{\log(pqm)/n}$, typical of high-dimensional settings, and depends similarly on the same sparsity and grouping structures. The third inequality (27) bounds $M_{1}\left({\hat{B}}_{i}\right)$, a measure of the model complexity or effective sparsity of the estimator. This ensures that the estimated model is not overly dense and depends on the maximum eigenvalue $\psi_{\max}$.

## 4. Simulations

To test our method, we simulated data from model (1) with k=2, m=3, and (n,p)={(100,100),(100,200),(100,500),(200,200),(200,500),(200,1000)}. For each platform *i*, the design matrix $X_{i}$ was generated with entries drawn independently from a *p*-variate normal distribution with mean zero and unit standard deviation. To introduce high correlation within each row of the error matrix $E_{i}$, we set the diagonal elements of its covariance matrix $\Sigma_{i}$ to 1 and the off-diagonal elements to 0.8. The coefficient matrix $B_{i}$ was constructed with 10 active rows. Entries in these rows were sampled uniformly, either from U(−1.5,−0.5) or U(0.5,1.5).

The evaluation metrics encompass three primary aspects: parameter estimation accuracy across all platforms, prediction accuracy, and feature selection accuracy. For each platform i=1,…,k, we measure the Frobenius norm of the deviation between the true regression coefficient matrix $B_{i}^{*}$ and its estimate ${\hat{B}}_{i}$, written as follows:

$$B_{F,i}={∥B_{i}^{*}-{\hat{B}}_{i}∥}_{F},$$

the deviation between the true covariance matrix $\Sigma_{i}^{*}$ and its estimate ${\hat{\Sigma }}_{i}$,

$$\Sigma_{F,i}={∥\Sigma_{i}^{*}-{\hat{\Sigma }}_{i}∥}_{F},$$

and the root mean squared errors (RMSE), i.e.,

$${\mathrm{RMSE}}_{i}={∥X_{i}B_{i}^{*}-X_{i}{\hat{B}}_{i}∥}_{F}/\sqrt{n},$$

where $X_{i}$ is out of samples for all platforms. The overall estimation accuracy of B and Σ can be summarized by the average across all *k* platforms, written as follows:

$$\bar{B_{F}}=\frac{1}{k}\sum_{i=1}^{k}B_{F,i},\;\bar{\Sigma_{F}}=\frac{1}{k}\sum_{i=1}^{k}\Sigma_{F,i},\;\mathrm{and}\;\bar{\mathrm{RMSE}}=\frac{1}{k}\sum_{i=1}^{k}{\mathrm{RMSE}}_{i}.$$

Meanwhile, feature selection performance is evaluated using sensitivity, the proportion of truly active predictors that are correctly identified, written as follows:

$$\mathrm{Sensitivity}=\frac{TP}{TP+FN},$$

and specificity, the proportion of truly inactive predictors that are correctly excluded,

$$\mathrm{Specificity}=\frac{TN}{TN+FP},$$

where *TP* and *TN* represent the number of predictors correctly identified as active (non-zero true coefficients with non-zero estimates) and inactive (zero true coefficients with zero estimates), respectively. Conversely, *FN* and *FP* denote the number of predictors incorrectly identified as active (zero true coefficients with non-zero estimates) and inactive (non-zero true coefficients with zero estimates), respectively. These metrics are computed for each platform, and the results are aggregated across all *k* platforms to provide a comprehensive assessment of feature selection performance.

We compare our proposed method with two alternative approaches, FusionLearn and multivariate Group Lasso. The FusionLearn method, implemented in the R package FusionLearn [6], is designed for multi-platform data integration but treats responses as univariate. To adapt it to our multivariate setting, we apply it separately to each of the *m* responses across platforms, which ignores the covariance structure among responses. Conversely, the multivariate Group Lasso method, available via the glmnet package [19], is designed for multivariate regression within a single platform. We therefore apply it independently to the data from each platform, which does not leverage information shared across platforms.

The simulation results are given in Table 2 and Table 3. As is seen in Table 2, the comparison of MM-HLR, FusionLearn, and GroupLasso reveals distinct performance in parameter estimation and prediction. In terms of parameter estimation of B and Σ, the proposed MM-HLR consistently provides estimates closest to the true ones, reflected by their smallest measures. In contrast, both FusionLearn and GroupLasso systematically overestimate the bias of parameters. The estimates of B and Σ via GroupLasso are often excessively high, especially for smaller *n* and larger *p*. In terms of prediction performance, MM-HLR demonstrates a strong advantage, achieving the lowest RMSE in every (n,p) setting. Its RMSE values range from 0.38 to 0.58, which are much lower than those of the other two methods. These consistent superiorities confirm MM-HLR’s excellent parameter estimation and predictive capability.

Table 3 presents the comparison of averaged feature selection accuracy (sensitivity and specificity) across different methods under various (n,p) configurations. All three methods achieve perfect sensitivity (1.0) across all pairs of (n,p), indicating their superior ability to select true predictors. However, the specificity values vary significantly among the three methods. MM-HLR achieves near-perfect or perfect specificity (0.9996 to 1.0) in all scenarios. GroupLasso has the lowest specificity overall, ranging from 0.7362 to 0.9396, indicating its weaker ability to exclude irrelevant predictors than the other two methods, while the ability of FusionLearn to exclude irrelevant predictors is in the middle, exhibiting the specificity values from 0.9166 to 0.9524.

## 5. Real Data Analysis

Zhang et al. [7] integrated three financial indices (S&P 500, Dow Jones, and VIX) as distinct platforms. Similarly, we consider a two-platform analysis (K=2) of U.S. stock market data. Our first platform comprises the S&P 500 index and its volatility index (VIX), while the second consists of the NASDAQ-100 index and its volatility index (VXN). Thus, each platform provides a two-dimensional response vector (m=2). We consider stocks that are actively traded in either the S&P 500 or NASDAQ-100 indices as predictors. The analysis uses weekly data from October 2022 to September 2025. To achieve approximately independent samples, log returns are calculated for all series. After data preprocessing and the removal of missing values, the final dataset contains 156 weekly observations and 510 predictors, which are common to both platforms. The correlations between two responses within each platform are −0.79 for Platform 1 (S&P 500 and VIX) and −0.66 for Platform 2 (NASDAQ-100 and VXN). The dataset is randomly partitioned into a training set of 100 samples (n=100) and a test set comprising the remaining observations ($n_{test}=56$). Our objective is to identify a common set of predictors that are relevant to at least one of the responses in either platform.

Table 4 presents the prediction errors on the test samples for Platform *i* $\left(PE_{i},\;i=1,2\right)$ and their average (PE), where $PE_{i}={∥Y_{i}-X_{i}{\hat{B}}_{i}∥}_{F}/\sqrt{n_{\mathrm{test}}}$. We also report the number of selected predictors (support) to quantify the sparsity of the estimated model. The proposed MM-HLR method demonstrates superior overall performance compared to the benchmark methods, FusionLearn and GroupLasso, in terms of both prediction accuracy and model sparsity. Specifically, MM-HLR achieves the lowest total prediction error (PE=0.0828), which is much lower than that of FusionLearn (PE=0.8903) and approximately 20% lower than that of GroupLasso (PE=0.1067). This advantage is consistent across both individual platforms. Furthermore, MM-HLR yields the most parsimonious model, selecting only 6 predictors. In contrast, FusionLearn and GroupLasso select 115 and 75 predictors, respectively, suggesting a higher risk of overfitting. This effective balance between prediction accuracy and model simplicity underscores the capability of the MM-HLR method to leverage both multivariate dependence and multi-platform structures within high-dimensional regression.

## 6. Conclusions and Discussion

In this paper, we introduce the multi-platform high-dimensional multivariate linear regression model, designed to simultaneously model correlated multivariate responses across multiple platforms. The proposed framework explicitly accounts for within-platform response correlation while incorporating a group Lasso penalty to fuse information across platforms, thereby promoting both individual sparsity and structured grouping of predictors. The optimization algorithm, combining iteratively reweighted least squares with block coordinate descent, is developed to solve the resulting regularization problem efficiently. Theoretical guarantees are established for the estimator $\hat{B}$, covering prediction accuracy, estimation error bounds, and support recovery (sparsity) under regularity conditions. Simulation studies under various scenarios demonstrate the outperformance of MM-HLR in parameter estimation, prediction, and variable selection, showing minimal bias, low variance, and strong robustness across all tested conditions. The superior performance of our approach is further validated through an analysis of real financial data, which confirms its effectiveness in leveraging both multivariate dependence and multi-platform structures within high-dimensional regression.

In this article, we impose a shared sparsity structure across platforms. Extending the framework to handle scenarios with only partial overlap would be a valuable direction for future work. Accordingly, we note that the method’s performance may degrade if the common sparsity pattern is violated and that formally relaxing this assumption constitutes an important avenue for future research.

## Figures and Tables

**Table 1:** Data structure of response matrix $Y_{i}\in R^{n\times m}$, design matrix $X_{i}\in R^{n\times p}$ and parameter matrix $B_{i}\in R^{p\times m}$ for platform *i*, where $y_{ijs}$ and $X_{ijr}$ denote the *j*-th observed value of response *s* and predictor *r* on platform *i*, respectively. The corresponding regression coefficient of predictor *r* on response *s* is $\beta_{irs}$, i=1,…,k; j=1,…,n; s=1,…,m; r=1,…,p.

	Platform 1	…	Platform *k*
Parameter matrix	$B_{1}=\left(\begin{array}{ccc} \theta_{111} & \ldots & \theta_{11m}\\ \vdots & \ddots & \vdots \\ \theta_{1p1} & \ldots & \theta_{1pm} \end{array}\right)$	…	$B_{k}=\left(\begin{array}{ccc} \theta_{k11} & \ldots & \theta_{k1m}\\ \vdots & \ddots & \vdots \\ \theta_{kp1} & \ldots & \theta_{kpm} \end{array}\right)$
Response matrix	$Y_{1}=\left(\begin{array}{ccc} Y_{111} & \ldots & Y_{11m}\\ \vdots & \ddots & \vdots \\ Y_{1n1} & \ldots & Y_{1nm} \end{array}\right)$	…	$Y_{k}=\left(\begin{array}{ccc} Y_{k11} & \ldots & Y_{k1m}\\ \vdots & \ddots & \vdots \\ Y_{kn1} & \ldots & Y_{knm} \end{array}\right)$
Design matrix	$X_{1}=\left(\begin{array}{ccc} X_{111} & \ldots & X_{11p}\\ \vdots & \ddots & \vdots \\ X_{1n1} & \ldots & X_{1np} \end{array}\right)$	…	$X_{k}=\left(\begin{array}{ccc} X_{k11} & \ldots & X_{k1p}\\ \vdots & \ddots & \vdots \\ X_{kn1} & \ldots & X_{knp} \end{array}\right)$

**Table 2:** Comparison of averaged parameter estimation accuracy ($B_{F},\Sigma_{F}$) and prediction accuracy (RMSE) across different methods under various (n,p) configurations (standard errors in parentheses).

(*n*, *p*)	Methods	$B_{F,1}$	$B_{F,2}$	$\bar{B_{F}}$	RMSE_1_	RMSE_2_	$\bar{\mathrm{RMSE}}$	$\Sigma_{F,1}$	$\Sigma_{F,2}$	$\bar{\Sigma_{F}}$
(100, 100)	MM-HLR	0.5653	0.5830	0.5742	0.5598	0.5719	0.5659	0.4486	0.4328	0.4407
		(0.13)	(0.13)	(0.09)	(0.13)	(0.11)	(0.09)	(0.24)	(0.18)	(0.13)
	FusionLearn	0.7115	0.7485	0.7300	0.7065	0.7444	0.7254	0.5298	0.5157	0.5228
		(0.11)	(0.13)	(0.08)	(0.11)	(0.12)	(0.08)	(0.24)	(0.19)	(0.15)
	GroupLasso	1.0402	1.0629	1.0516	1.0522	1.0782	1.0652	0.9649	1.0862	1.0256
		(0.13)	(0.14)	(0.08)	(0.14)	(0.14)	(0.09)	(0.41)	(0.36)	(0.30)
(100, 200)	MM-HLR	0.5821	0.5808	0.5814	0.5770	0.5764	0.5767	0.4282	0.4152	0.4217
		(0.13)	(0.13)	(0.09)	(0.13)	(0.13)	(0.10)	(0.24)	(0.24)	(0.17)
	FusionLearn	0.7494	0.7583	0.7539	0.7437	0.7463	0.7450	0.6083	0.5350	0.5717
		(0.11)	(0.15)	(0.08)	(0.11)	(0.14)	(0.09)	(0.23)	(0.21)	(0.15)
	GroupLasso	1.1937	1.1821	1.1879	1.1925	1.1590	1.1757	1.2531	1.3192	1.2862
		(0.16)	(0.15)	(0.11)	(0.17)	(0.16)	(0.12)	(0.48)	(0.46)	(0.33)
(100, 500)	MM-HLR	0.5594	0.5944	0.5769	0.5566	0.5864	0.5715	0.3718	0.4083	0.3901
		(0.11)	(0.12)	(0.09)	(0.12)	(0.12)	(0.09)	(0.20)	(0.23)	(0.15)
	FusionLearn	0.7646	0.7613	0.7629	0.7643	0.7593	0.7618	0.8426	0.7951	0.8189
		(0.11)	(0.09)	(0.08)	(0.12)	(0.10)	(0.08)	(0.20)	(0.19)	(0.12)
	GroupLasso	1.2484	1.2021	1.2252	1.2593	1.1910	1.2252	1.6113	1.6055	1.6084
		(0.15)	(0.14)	(0.11)	(0.17)	(0.15)	(0.11)	(0.49)	(0.47)	(0.38)
(200, 200)	MM-HLR	0.3998	0.3861	0.3930	0.3982	0.3864	0.3923	0.2941	0.2429	0.2685
		(0.08)	(0.08)	(0.06)	(0.08)	(0.08)	(0.06)	(0.15)	(0.12)	(0.10)
	FusionLearn	0.5087	0.5190	0.5138	0.5062	0.5181	0.5122	0.3608	0.3300	0.3454
		(0.09)	(0.07)	(0.06)	(0.09)	(0.07)	(0.06)	(0.17)	(0.14)	(0.11)
	GroupLasso	0.7809	0.7788	0.7798	0.7768	0.7678	0.7723	0.6529	0.6957	0.6743
		(0.08)	(0.08)	(0.05)	(0.09)	(0.07)	(0.05)	(0.31)	(0.28)	(0.20)
(200, 500)	MM-HLR	0.3845	0.3871	0.3858	0.3828	0.3857	0.3843	0.2695	0.2657	0.2676
		(0.08)	(0.07)	(0.05)	(0.08)	(0.08)	(0.05)	(0.13)	(0.17)	(0.10)
	FusionLearn	0.5516	0.5473	0.5495	0.5511	0.5428	0.5469	0.5278	0.5431	0.5355
		(0.07)	(0.09)	(0.05)	(0.07)	(0.09)	(0.05)	(0.17)	(0.17)	(0.11)
	GroupLasso	0.8847	0.8629	0.8738	0.8806	0.8539	0.8673	1.0466	1.0352	1.0409
		(0.10)	(0.10)	(0.06)	(0.10)	(0.10)	(0.07)	(0.41)	(0.42)	(0.32)
(200, 1000)	MM-HLR	0.4068	0.4044	0.4056	0.4084	0.3985	0.4035	0.2506	0.2579	0.2542
		(0.09)	(0.09)	(0.06)	(0.09)	(0.08)	(0.06)	(0.14)	(0.15)	(0.10)
	FusionLearn	0.5893	0.5762	0.5827	0.5893	0.5707	0.5800	0.6546	0.6876	0.6711
		(0.09)	(0.10)	(0.07)	(0.09)	(0.09)	(0.07)	(0.16)	(0.15)	(0.10)
	GroupLasso	0.9324	0.9642	0.9474	0.9336	0.9592	0.9464	1.1231	1.0740	1.0985
		(0.10)	(0.11)	(0.08)	(0.11)	(0.11)	(0.09)	(0.44)	(0.48)	(0.35)

**Table 3:** Comparison of averaged feature selection accuracy (sensitivity and specificity) across different methods under various (n,p) configurations (standard errors in parentheses).

(*n*, *p*)	Sensitivity			Specifitity		
	MM-HLR	FusionLearn	GroupLasso	MM-HLR	FusionLearn	GroupLasso
(100, 100)	1.0000(0.00)	1.0000(0.00)	1.0000(0.00)	0.9996(0.00)	0.9166(0.02)	0.7362(0.08)
(100, 200)	1.0000(0.00)	1.0000(0.00)	1.0000(0.00)	0.9996(0.00)	0.9257(0.02)	0.8152(0.06)
(100, 500)	1.0000(0.00)	1.0000(0.00)	1.0000(0.00)	1.0000(0.00)	0.9496(0.01)	0.8944(0.04)
(200, 200)	1.0000(0.00)	1.0000(0.00)	1.0000(0.00)	1.0000(0.00)	0.9326(0.02)	0.8392(0.05)
(200, 500)	1.0000(0.00)	1.0000(0.00)	1.0000(0.00)	0.99996(0.00)	0.9394(0.01)	0.8962(0.04)
(200, 1000)	1.0000(0.00)	1.0000(0.00)	1.0000(0.00)	1.0000(0.00)	0.9524(0.01)	0.9396(0.02)

**Table 4:** The platform-specific prediction errors $\left(PE_{i},\;i=1,2\right)$, the average prediction error (PE), and the number of selected predictors (*support*).

Method	*PE*_1_	*PE*_2_	*PE*	*Support*
MM-HLR	0.0993	0.0663	0.0828	6
FusionLearn	0.8895	0.8912	0.8903	91
GroupLasso	0.1257	0.0877	0.1067	75

## Data Availability

The original contributions presented in this study are included in the article. Further inquiries can be directed to the corresponding author.

## Appendix A. Theoretical Proofs

We provide the proof of Theorem 1. Before proceeding, we present Lemmas A1 and A2, which are required in the proof of Theorem 1.

**Lemma** **A1.**
*Define a random matrix $W_{i}=E_{i}\Sigma_{i}^{-1}\in R^{n\times m}$ and a random variable $V_{irs}=X_{i}^{(r)⊤}W_{i}^{\left(s\right)}$, where $W_{i}^{\left(s\right)}\in R^{n}$ is the sth column of $W_{i}$, s=1,…,m; i=1,…,k. Define an event A and its complementary event $A^{c}$, respectively, as follows:*

$$A=\{||X_{i}^{⊤}W_{i}|{|}_{\infty }\leq \frac{n\lambda }{2},\mathrm{for}\;\mathrm{all}\;i=1,\ldots ,k\},$$

$$A^{c}=\left\{\mathrm{at}\;\mathrm{least}\;\mathrm{one}\;\right|V_{irs}\left|\geq \frac{n\lambda }{2},i=1,\ldots ,k,r=1,\ldots ,p,s=1,\ldots ,m\right\}.$$

*By Assumptions 1 and 2, the following holds:*

$$Pr\left\{A^{c}\right\}\leq {\left(pmk\right)}^{1-A^{2}/2}\to 0,$$

*for some constant $A>\sqrt{2}$.*

**Proof** **of Lemma A1.** Since the rows of $E_{i}$ follows $N_{m}\left(0,\Sigma_{i}^{*}\right)$ independently and identically (iid), then the rows of $W_{i}$ are iid with $N_{m}\left(0,\Sigma_{i}^{-1}\Sigma_{i}^{*}\Sigma_{i}^{-1}\right)$ random vectors. For the covariance matrix $\Sigma_{i}^{-1}\Sigma_{i}^{*}\Sigma_{i}^{-1}$, by Assumption 2, we have the following:

$$\begin{array}{c} -\lambda^{*}I⪯\Sigma_{i}-\Sigma_{i}^{*}⪯\lambda^{*}I,\Sigma^{*}⪯C_{2}I\Rightarrow \left(C_{1}-\lambda^{*}\right)I⪯\Sigma_{i}⪯\left(\lambda^{*}+C_{2}\right)I\\ \Rightarrow \Sigma_{i}^{-1}\Sigma_{i}^{*}\Sigma_{i}^{-1}⪯\Sigma_{i}^{-1}+\lambda^{*}\Sigma_{i}^{-2}⪯\left(\frac{1}{C_{1}-\lambda^{*}}+\frac{\lambda^{*}}{{\left(C_{1}-\lambda^{*}\right)}^{2}}\right)I=\frac{C_{1}}{{\left(C_{1}-\lambda^{*}\right)}^{2}}I, \end{array}$$

which means that the diagonal elements of $\Sigma_{i}^{-1}\Sigma_{i}^{*}\Sigma_{i}^{-1}$ are less than $\sigma^{2}≜\frac{C_{1}}{{\left(C_{1}-\lambda^{*}\right)}^{2}}$. Since $W_{i}^{\left(s\right)}∼N_{n}\left(0,\sigma_{is}^{2}I\right)$, where $\sigma_{is}^{2}$ is the *s*th diagonal element of $\Sigma_{i}^{-1}\Sigma_{i}^{*}\Sigma_{i}^{-1}$. By Assumption 1, $Var\left(V_{irs}\right)=X_{i}^{(r)⊤}cov\left(W_{i}^{\left(s\right)}\right)X_{i}^{\left(r\right)}=n\sigma_{is}^{2}\leq n\sigma$. Therefore ${\left(n\sigma_{is}^{2}\right)}^{-\frac{1}{2}}V_{irs}≜Z$ are standard normal random variables for all i,r,s. We have the following:

$$\begin{array}{ccc} Pr\{A^{c}\} & \leq & \sum_{r=1}^{p}\sum_{s=1}^{m}\sum_{i=1}^{k}Pr\{|V_{irs}\left|\geq \frac{n\lambda }{2}\right\}\\ & = & p\sum_{s=1}^{m}\sum_{i=1}^{k}Pr\{|V_{irs}\left|/\sqrt{n}\sigma_{is}\geq \frac{\sqrt{n}\lambda }{2\sigma_{is}}\right\}\\ & \leq & p\sum_{s=1}^{m}\sum_{i=1}^{k}Pr\{|Z|\geq \frac{\sqrt{n}\lambda }{2\sigma }\}\\ & \leq & pmk\exp\left(-\frac{\lambda^{2}n}{8\sigma^{2}}\right)={\left(pmk\right)}^{1-A^{2}/2}, \end{array}$$

where $\lambda =2\sigma A{\left\{\log\left(pmk\right)/n\right\}}^{1/2}$. □

**Lemma** **A2.**
*Under Assumptions 1–3, for any $B_{i}\in R^{p\times m},i=1,\ldots ,k$, with probability at least $1-{\left(pmk\right)}^{1-A^{2}/2}$, the following holds:*

$$\begin{array}{cc} \frac{1}{n}\sum_{i=1}^{k}\mathrm{tr}\left[\left(X_{i}\left(B_{i}^{*}-{\hat{B}}_{i}\right)\right)\Sigma_{i}^{-1}{\left(X_{i}\left(B_{i}^{*}-{\hat{B}}_{i}\right)\right)}^{⊤}\right]+\lambda \sum_{i=1}^{k}\left|\right|{\hat{B}}_{i}-B_{i}{\left|\right|}_{1}+2\sum_{r=1}^{p}\lambda_{g}\left|\right|{\hat{\Theta }}^{\left(r\right)}-\Theta^{\left(r\right)}{\left|\right|}_{F} & \; \end{array}$$

(A1)
$$\begin{array}{cc} \leq & \frac{1}{n}\sum_{i=1}^{k}\mathrm{tr}\left[\left(X_{i}\left(B_{i}^{*}-B_{i}\right)\right)\Sigma_{i}^{-1}{\left(X_{i}\left(B_{i}^{*}-B_{i}\right)\right)}^{⊤}\right]+4\lambda \sum_{i=1}^{k}\sum_{rs\in J_{1}\left(B_{i}\right)}|{\hat{\theta }}_{irs}-\theta_{irs}|+4\sum_{r\in J_{2}\left(B\right)}\lambda_{g}\left|\right|{\hat{\Theta }}^{\left(r\right)}-\Theta^{\left(r\right)}{\left|\right|}_{F}\\ \mathrm{and} \end{array}$$

(A2)
$$\begin{array}{cc} M_{1}\left(\hat{B_{i}}\right)\leq \frac{4}{\lambda^{2}n^{2}}\left|\right|X_{i}^{⊤}X_{i}\left(\hat{B_{i}}-B_{i}^{*}\right)\Sigma_{i}^{-1}{\left|\right|}_{F}^{2}. & \end{array}$$

**Proof** **of Lemma A2.** For any $B_{i}\in R^{p\times m},i=1,\ldots ,k$, $Q\left(\hat{B}\right)\leq Q\left(B\right)$. Plugging $Y_{i}=X_{i}B_{i}^{*}+E_{i},\;i=1,\ldots ,k$ into the above inequality, results in the following:

$$\begin{array}{cc} \; & \frac{1}{n}\sum_{i=1}^{k}\mathrm{tr}\left[\left(X_{i}\left(B_{i}^{*}-{\hat{B}}_{i}\right)\right)\Sigma_{i}^{-1}{\left(X_{i}\left(B_{i}^{*}-{\hat{B}}_{i}\right)\right)}^{⊤}\right] \end{array}$$

$$\begin{array}{c} \leq \frac{1}{n}\sum_{i=1}^{k}\mathrm{tr}\left[\left(X_{i}\left(B_{i}^{*}-B_{i}\right)\right)\Sigma_{i}^{-1}{\left(X_{i}\left(B_{i}^{*}-B_{i}\right)\right)}^{⊤}\right] \end{array}$$

$$\begin{array}{cc} +\frac{2}{n}\sum_{i=1}^{k}\left|\right|X_{i}^{⊤}E_{i}\Sigma_{i}^{-1}{\left|\right|}_{\infty }\left|\right|{\hat{B}}_{i}-B_{i}{\left|\right|}_{1} & \; \end{array}$$

(A3) $$\begin{array}{cc} \; & +2\lambda \sum_{i,r,s}\left(\right|\theta_{irs}\left|-\right|{\hat{\theta }}_{irs}\left|\right)+2\sum_{r=1}^{p}\lambda_{g}\left(|\right|\Theta^{\left(r\right)}{\left|\right|}_{F}-\left|\right|{\hat{\Theta }}^{(r)⊤}{\left|\right|}_{F}). \end{array}$$

By Lemma A1, it holds that on event A, the following is calculated:

$$\begin{array}{cc} \; & \frac{1}{n}\sum_{i=1}^{k}\mathrm{tr}\left[\left(X_{i}\left(B_{i}^{*}-{\hat{B}}_{i}\right)\right)\Sigma_{i}^{-1}{\left(X_{i}\left(B_{i}^{*}-{\hat{B}}_{i}\right)\right)}^{⊤}\right]+\lambda \sum_{i=1}^{k}\left|\right|{\hat{B}}_{i}-B_{i}{\left|\right|}_{1}+2\sum_{r=1}^{m}\lambda_{g}\left|\right|{\hat{\Theta }}^{\left(r\right)}-\Theta^{\left(r\right)}{\left|\right|}_{F} \end{array}$$

$$\begin{array}{cc} \leq \frac{1}{n}\sum_{i=1}^{k}\mathrm{tr}\left[\left(X_{i}\left(B_{i}^{*}-B_{i}\right)\right)\Sigma_{i}^{-1}{\left(X_{i}\left(B_{i}^{*}-B_{i}\right)\right)}^{⊤}\right]+2\lambda \sum_{i=1}^{k}\left(|\right|{\hat{B}}_{i}-B_{i}{\left|\right|}_{1}+\left|\right|B_{i}{\left|\right|}_{1}-\left|\right|{\hat{B}}_{i}{\left|\right|}_{1}) & \; \end{array}$$

$$\begin{array}{cc} +2\sum_{r=1}^{p}\lambda_{g}\left(|\right|{\hat{\Theta }}^{\left(r\right)}-\Theta^{\left(r\right)}{\left|\right|}_{F}+\left|\right|\Theta^{\left(r\right)}{\left|\right|}_{F}-\left|\right|{\hat{\Theta }}^{\left(r\right)}{\left|\right|}_{F}) & \; \end{array}$$

$$\begin{array}{cc} \leq \frac{1}{n}\sum_{i=1}^{k}\mathrm{tr}\left[\left(X_{i}\left(B_{i}^{*}-B_{i}\right)\right)\Sigma_{i}^{-1}{\left(X_{i}\left(B_{i}^{*}-B_{i}\right)\right)}^{⊤}\right]+4\lambda \sum_{i=1}^{k}\sum_{rs\in J_{1}\left(B\right)}\left|{\hat{\theta }}_{irs}-\theta_{irs}\right| & \; \end{array}$$

(A4) $$\begin{array}{ccc} \; & +4\sum_{r\in J_{2}\left(B\right)}\lambda_{g}\left|\right|{\hat{\Theta }}^{\left(r\right)}-\Theta^{\left(r\right)}{\left|\right|}_{F}. & \; \end{array}$$

This completes the proof of the first inequality (A1) in Lemma A2. To prove the second inequality (A2) in Lemma A2, we use the KKT conditions. For $\forall \;{\hat{\theta }}_{irs}\neq 0$, the following is written:

$$X_{i}^{(r)⊤}\left(Y_{i}-X_{i}{\hat{B}}_{i}\right){\left(\Sigma_{i}^{-1}\right)}_{\cdot s}=2n\lambda sign\left({\hat{\theta }}_{irs}\right)+\frac{2n\lambda_{g}{\hat{\theta }}_{irs}}{\left|\right|{\hat{\Theta }}^{\left(r\right)}{\left|\right|}_{F}},$$

where ${\left(\Sigma_{i}^{-1}\right)}_{\cdot s}$ is the *s* column of $\Sigma_{i}^{-1}$, which implies that

(A5) $$\begin{array}{c} \lambda \leq \frac{1}{n}\left|X_{i}^{(r)⊤}\left(Y_{i}-X_{i}{\hat{B}}_{i}\right){\left(\Sigma_{i}^{-1}\right)}_{\cdot s}\right|. \end{array}$$

On the other hand, we have on event A, written as follows:

$$\begin{array}{ccc} \frac{1}{n}\left|X_{i}^{(r)⊤}\left(Y_{i}-X_{i}{\hat{B}}_{i}\right){\left(\Sigma_{i}^{-1}\right)}_{\cdot s}\right| & \leq & \frac{1}{n}\left|X_{i}^{(r)⊤}X_{i}\left(B_{i}^{*}-{\hat{B}}_{i}\right){\left(\Sigma_{i}^{-1}\right)}_{\cdot s}+X_{i}^{(r)⊤}E_{i}{\left(\Sigma_{i}^{-1}\right)}_{\cdot s}\right| \end{array}$$

$$\begin{array}{cccc} & \; & \leq & \frac{1}{n}|X_{i}^{(r)⊤}X_{i}\left(B_{i}^{*}-{\hat{B}}_{i}\right){\left(\Sigma_{i}^{-1}\right)}_{\cdot s}|+\frac{1}{n}\left|\right|X_{i}^{⊤}W_{i}{\left|\right|}_{\infty } \end{array}$$

(A6) $$\begin{array}{ccc} \; & \leq & \frac{1}{n}\left|X_{i}^{(r)⊤}X_{i}\left(B_{i}^{*}-{\hat{B}}_{i}\right){\left(\Sigma_{i}^{-1}\right)}_{\cdot s}\right|+\frac{\lambda }{2}. \end{array}$$

Combining Equations (A5) and (A6) together, we can obtain the following:

$$\frac{\lambda }{2}\leq \frac{1}{n}\left|X_{i}^{(r)⊤}X_{i}\left(B_{i}^{*}-{\hat{B}}_{i}\right){\left(\Sigma_{i}^{-1}\right)}_{\cdot s}\right|.$$

Therefore, for any platform *i*, the following is written:

$$\begin{array}{cc} M_{1}\left(\hat{B_{i}}\right) & \leq \frac{4}{\lambda^{2}n^{2}}\sum_{rs\in J_{1}\left({\hat{B}}_{i}\right)}{\left|X_{i}^{(r)⊤}X_{i}\left(B_{i}^{*}-{\hat{B}}_{i}\right){\left(\Sigma_{i}^{-1}\right)}_{\cdot s}\right|}^{2} \end{array}$$

(A7) $$\begin{array}{c} \leq \frac{4}{\lambda^{2}n^{2}}\left|\right|X_{i}^{⊤}X_{i}\left(B_{i}^{*}-{\hat{B}}_{i}\right)\Sigma_{i}^{-1}{\left|\right|}_{F}^{2}. \end{array}$$

This completes the proof of Lemma A2. □

Now, we detail the proof of Theorem 1.

**Proof** **of Theorem 1.** By the first inequality (A1) in Lemma A2 and letting $B=B^{*}$, we have, on event A, the following:

$$\begin{array}{cc} \frac{1}{n}\sum_{i=1}^{k}\mathrm{tr}\left[\left(X_{i}\left(B_{i}^{*}-{\hat{B}}_{i}\right)\right)\Sigma_{i}^{-1}{\left(X_{i}\left(B_{i}^{*}-{\hat{B}}_{i}\right)\right)}^{⊤}\right] & \; \end{array}$$

$$\begin{array}{cc} \leq 4\lambda \sum_{i=1}^{k}\sum_{rs\in J_{1}\left(B_{i}^{*}\right)}|{\hat{\theta }}_{irs}-\theta_{irs}^{*}|+4\sum_{r\in J_{2}\left(B^{*}\right)}\lambda_{g}\left|\right|{\hat{\Theta }}^{\left(r\right)}-\Theta^{(r)*}{\left|\right|}_{F} & \; \end{array}$$

(A8) $$\begin{array}{cc} \; & \leq 4\lambda u^{\frac{1}{2}}\sum_{i=1}^{k}\left|\right|{\left({\hat{B}}_{i}-B_{i}^{*}\right)}_{J_{1}\left(B_{i}^{*}\right)}{\left|\right|}_{F}+4{\left(\sum_{r\in J_{2}\left(B^{*}\right)}\lambda_{g}^{2}\right)}^{\frac{1}{2}}\left|\right|{\left(\hat{B}-B^{*}\right)}_{J_{2}\left(B^{*}\right)}{\left|\right|}_{F}, \end{array}$$

which is derived by Cauchy–Schwarz inequality. On event A, we also have the following:

$$\begin{array}{cc} \lambda \sum_{i=1}^{k}\left|\right|{\hat{B}}_{i}-B_{i}^{*}{\left|\right|}_{1}+2\sum_{r=1}^{p}\lambda_{g}\left|\right|{\hat{\Theta }}^{\left(r\right)}-\Theta^{(r)*}{\left|\right|}_{F} & \; \end{array}$$

(A9) $$\begin{array}{c} \leq 4\lambda \sum_{i=1}^{k}\sum_{rs\in J_{1}\left(B_{i}^{*}\right)}|{\hat{\theta }}_{irs}-\theta_{irs}^{*}|+4\sum_{r\in J_{2}\left(B^{*}\right)}\lambda_{g}\left|\right|{\hat{\Theta }}^{\left(r\right)}-\Theta^{(r)*}{\left|\right|}_{F}, \end{array}$$

which, together with the fact that

(A10) $$\begin{array}{ccc} \; & \left|\right|{\hat{B}}_{i}-B_{i}^{*}{\left|\right|}_{1}=\sum_{rs\in J_{1}\left(B_{i}^{*}\right)}|{\hat{\theta }}_{irs}-\theta_{irs}^{*}|+\sum_{rs\in J_{1}^{c}\left(B_{i}^{*}\right)}\left|{\hat{\theta }}_{irs}-\theta_{irs}^{*}\right|, & \; \end{array}$$

(A11) $$\begin{array}{cc} \; & \sum_{r=1}^{p}\left|\right|{\hat{\Theta }}^{\left(r\right)}-\Theta^{(r)*}{\left|\right|}_{F}=\sum_{r\in J_{2}\left(B^{*}\right)}\left|\right|{\hat{\Theta }}^{\left(r\right)}-\Theta^{(r)*}{\left|\right|}_{F}+\sum_{r\in J_{2}^{c}\left(B^{*}\right)}\left|\right|{\hat{\Theta }}^{\left(r\right)}-\Theta^{(r)*}{\left|\right|}_{F}, \end{array}$$

yield the following inequality,

$$\begin{array}{ccc} \; & \lambda \sum_{i=1}^{k}\sum_{rs\in J_{1}^{c}\left(B_{i}^{*}\right)}|{\hat{\theta }}_{irs}-\theta_{irs}^{*}|+2\sum_{r\in J_{2}^{c}\left(B^{*}\right)}\lambda_{g}\left|\right|{\hat{\Theta }}^{\left(r\right)}-\Theta^{(r)*}{\left|\right|}_{F} & \; \end{array}$$

(A12) $$\begin{array}{cc} \; & \leq 3\lambda \sum_{i=1}^{k}\sum_{rs\in J_{1}\left(B_{i}^{*}\right)}|{\hat{\theta }}_{irs}-\theta_{irs}^{*}|+2\sum_{r\in J_{2}\left(B^{*}\right)}\lambda_{g}\left|\right|{\hat{\Theta }}^{\left(r\right)}-\Theta^{(r)*}{\left|\right|}_{F}. \end{array}$$

Thus the inequality (22) in Assumption 3 holds with $\Delta_{i}=\hat{B_{i}}-B_{i}^{*},i=1,\ldots ,k$ and $\rho_{r}=\lambda_{g}/\lambda$. Therefore, we obtain the following:

(A13) $$\begin{array}{c} \sum_{i=1}^{k}\left|\right|{\left(\hat{B_{i}}-B_{i}^{*}\right)}_{J_{1}\left(B^{*}\right)}{\left|\right|}_{F}\leq \frac{\sum_{i=1}^{k}{\left(\mathrm{tr}[\left(X_{i}\left(B_{i}^{*}-{\hat{B}}_{i}\right)\right)\Sigma_{i}^{-1}{\left(X_{i}\left(B_{i}^{*}-{\hat{B}}_{i}\right)\right)}^{⊤}]\right)}^{\frac{1}{2}}}{n^{1/2}\kappa_{1}}, \end{array}$$

(A14) $$\begin{array}{c} \left|\right|{\left(\hat{B}-B^{*}\right)}_{J_{2}\left(B^{*}\right)}{\left|\right|}_{F}\leq \frac{\sum_{i=1}^{k}{\left(\mathrm{tr}[\left(X_{i}\left(B_{i}^{*}-{\hat{B}}_{i}\right)\right)\Sigma_{i}^{-1}{\left(X_{i}\left(B_{i}^{*}-{\hat{B}}_{i}\right)\right)}^{⊤}]\right)}^{\frac{1}{2}}}{n^{1/2}\kappa_{2}}. \end{array}$$

Plugging Equations (A13) and (A14) into (A8), we have the following:

$$\begin{array}{cc} \frac{1}{n}\sum_{i=1}^{k}\mathrm{tr}\left[\left(X_{i}\left(B_{i}^{*}-{\hat{B}}_{i}\right)\right)\Sigma_{i}^{-1}{\left(X_{i}\left(B_{i}^{*}-{\hat{B}}_{i}\right)\right)}^{⊤}\right] & \; \end{array}$$

$$\begin{array}{cc} \; & \leq \left(\frac{4\lambda u^{\frac{1}{2}}}{n^{\frac{1}{2}}\kappa_{1}}+\frac{4{\left(\sum_{r\in J_{2}\left(B^{*}\right)}\lambda_{g}^{2}\right)}^{\frac{1}{2}}}{n^{\frac{1}{2}}\kappa_{2}}\right)\sum_{i=1}^{k}{\left(\mathrm{tr}[X_{i}\left(B_{i}^{*}-{\hat{B}}_{i}\right)\Sigma_{i}^{-1}{\left(X_{i}\left(B_{i}^{*}-{\hat{B}}_{i}\right)\right)}^{⊤}]\right)}^{\frac{1}{2}} \end{array}$$

(A15) $$\begin{array}{cc} =\left(\frac{4\lambda u^{\frac{1}{2}}}{n^{\frac{1}{2}}\kappa_{1}}+\frac{4\lambda s_{{\tilde{\rho }}^{*}}^{\frac{1}{2}}}{n^{\frac{1}{2}}\kappa_{2}}\right)\sum_{i=1}^{k}{\left(\mathrm{tr}[X_{i}\left(B_{i}^{*}-{\hat{B}}_{i}\right)\Sigma_{i}^{-1}{\left(X_{i}\left(B_{i}^{*}-{\hat{B}}_{i}\right)\right)}^{⊤}]\right)}^{\frac{1}{2}}, & \; \end{array}$$

which yields that, by Cauchy–Schwarz inequality,

(A16) $$\begin{array}{c} \frac{1}{n}\sum_{i=1}^{k}\mathrm{tr}\left[\left(X_{i}\left(B_{i}^{*}-{\hat{B}}_{i}\right)\right)\Sigma_{i}^{-1}{\left(X_{i}\left(B_{i}^{*}-{\hat{B}}_{i}\right)\right)}^{⊤}\right]\leq 16\lambda^{2}k{\left(\frac{u^{\frac{1}{2}}}{\kappa_{1}}+\frac{s_{{\tilde{\rho }}^{*}}^{\frac{1}{2}}}{\kappa_{2}}\right)}^{2}. \end{array}$$

Next, we prove the second term (26) in Theorem 1. Define ${\left||B|\right|}_{2,1}=\sum_{i=1}^{k}\left|\right|B_{i}{\left|\right|}_{1}+\sum_{r=1}^{p}\left|\right|\Theta^{\left(r\right)}{\left|\right|}_{F}$. Hence, the following is calculated:

$$\left|\right|\hat{B}-B^{*}{\left|\right|}_{2,1}=\sum_{i=1}^{k}\left|\right|{\hat{B}}_{i}-B_{i}^{*}{\left|\right|}_{1}+\sum_{r=1}^{p}\left|\right|{\hat{\Theta }}^{\left(r\right)}-\Theta^{(r)*}{\left|\right|}_{F}\leq 2\sum_{i=1}^{k}\left|\right|{\hat{B}}_{i}-B_{i}^{*}{\left|\right|}_{1}.$$

Then we have the following:

$$\begin{array}{cc} \left(\lambda +\lambda \rho_{r}\right)\left|\right|\hat{B}-B^{*}{\left|\right|}_{2,1} & =\lambda ||\hat{B}-B^{*}{\left|\right|}_{2,1}+\rho_{r}\lambda ||\hat{B}-B^{*}{\left|\right|}_{2,1} \end{array}$$

$$\begin{array}{cc} \; & \leq 3\lambda \sum_{i=1}^{k}\left|\right|{\hat{B}}_{i}-B_{i}^{*}{\left|\right|}_{1}+\rho_{r}\lambda \sum_{r=1}^{p}\left|\right|{\hat{\Theta }}^{\left(r\right)}-\Theta^{(r)*}{\left|\right|}_{F} \end{array}$$

$$\begin{array}{cc} \; & \leq 3\lambda \sum_{i=1}^{k}\left|\right|{\hat{B}}_{i}-B_{i}^{*}{\left|\right|}_{1}+\sum_{r=1}^{p}\lambda_{g}\left|\right|{\hat{\Theta }}^{\left(r\right)}-\Theta^{(r)*}{\left|\right|}_{F} \end{array}$$

(A17) $$\begin{array}{cc} \; & \leq 3\left[\lambda \sum_{i=1}^{k}\left|\right|{\hat{B}}_{i}-B_{i}^{*}{\left|\right|}_{1}+2\sum_{r=1}^{p}\lambda_{g}\left|\right|{\hat{\Theta }}^{\left(r\right)}-\Theta^{(r)*}{\left|\right|}_{F}\right]. \end{array}$$

By (A9), we obtain the following:

$$\begin{array}{cc} \frac{1+\rho_{r}}{3}\lambda ||\hat{B}-B^{*}{\left|\right|}_{2,1} & \leq \lambda \sum_{i=1}^{k}\left|\right|{\hat{B}}_{i}-B_{i}^{*}{\left|\right|}_{1}+2\sum_{r=1}^{p}\lambda_{g}\left|\right|{\hat{\Theta }}^{\left(r\right)}-\Theta^{(r)*}{\left|\right|}_{F} \end{array}$$

$$\begin{array}{cc} \; & \leq 4\lambda \sum_{i=1}^{k}\sum_{rs\in J_{1}\left(B_{i}^{*}\right)}|{\hat{\theta }}_{irs}-\theta_{irs}^{*}|+4\sum_{r\in J_{2}\left(B^{*}\right)}\lambda_{g}\left|\right|{\hat{\Theta }}^{\left(r\right)}-\Theta^{(r)*}{\left|\right|}_{F} \end{array}$$

$$\begin{array}{cc} \; & \leq 4\lambda u^{1/2}\sum_{i=1}^{k}\left|\right|{\left({\hat{B}}_{i}-B_{i}^{*}\right)}_{J_{1}\left(B_{i}^{*}\right)}{\left|\right|}_{F}+4{\left(\sum_{r\in J_{2}\left(B^{*}\right)}\lambda_{g}^{2}\right)}^{1/2}\left|\right|{\left(\hat{B}-B^{*}\right)}_{J_{2}\left(B^{*}\right)}{\left|\right|}_{F} \end{array}$$

$$\begin{array}{cc} \; & \leq \left(\frac{4\lambda u^{1/2}}{n^{1/2}\kappa_{1}}+\frac{4\lambda s_{{\tilde{\rho }}^{*}}^{1/2}}{n^{1/2}\kappa_{2}}\right)\sum_{i=1}^{k}{\left(\mathrm{tr}[X_{i}\left(B_{i}^{*}-{\hat{B}}_{i}\right)\Sigma_{i}^{-1}{\left(X_{i}\left(B_{i}^{*}-{\hat{B}}_{i}\right)\right)}^{⊤}]\right)}^{1/2} \end{array}$$

$$\begin{array}{cc} \; & \leq \left(\frac{4\lambda u^{1/2}}{n^{1/2}\kappa_{1}}+\frac{4\lambda s_{{\tilde{\rho }}^{*}}^{1/2}}{n^{1/2}\kappa_{2}}\right)4{\left(kn\right)}^{1/2}\lambda \left(\frac{u^{1/2}}{\kappa_{1}}+\frac{s_{{\tilde{\rho }}^{*}}^{1/2}}{\kappa_{2}}\right) \end{array}$$

(A18) $$\begin{array}{cc} \; & =16k^{1/2}\lambda^{2}{\left(\frac{u^{1/2}}{\kappa_{1}}+\frac{s_{{\tilde{\rho }}^{*}}^{1/2}}{\kappa_{2}}\right)}^{2}. \end{array}$$

Therefore, we obtain the following:

(A19) $$\begin{array}{c} \sum_{i=1}^{k}\left|\right|{\hat{B}}_{i}-B_{i}^{*}{\left|\right|}_{1}\leq \frac{1}{2}\left|\right|\hat{B}-B^{*}{\left|\right|}_{2,1}\leq \frac{24\lambda k^{1/2}}{1+\rho }{\left(\frac{u^{1/2}}{\kappa_{1}}+\frac{s_{{\tilde{\rho }}^{*}}^{1/2}}{\kappa_{2}}\right)}^{2}. \end{array}$$

Finally, we prove the third term (27) in Theorem 1. By (A2) in Lemma A2, we can obtain the following:

(A20) $$\begin{array}{cc} M_{1}\left(\hat{B_{i}}\right) & =\frac{4}{\lambda^{2}n^{2}}\left|\right|X_{i}^{⊤}X_{i}\left(B_{i}^{*}-{\hat{B}}_{i}\right)\Sigma_{i}^{-1}{\left|\right|}_{F}^{2} \end{array}$$

$$\begin{array}{cc} \; & =\frac{4}{\lambda^{2}n^{2}}\mathrm{tr}\left(\Sigma_{i}^{-1}{\left(B_{i}^{*}-{\hat{B}}_{i}\right)}^{⊤}X_{i}^{⊤}X_{i}X_{i}^{⊤}X_{i}\left(B_{i}^{*}-{\hat{B}}_{i}\right)\Sigma_{i}^{-1}\right) \end{array}$$

$$\begin{array}{cc} \; & \leq \frac{4}{\lambda^{2}n}\psi_{\max}\mathrm{tr}\left(\Sigma_{i}^{-1}{\left(B_{i}^{*}-{\hat{B}}_{i}\right)}^{⊤}X_{i}^{⊤}X_{i}\left(B_{i}^{*}-{\hat{B}}_{i}\right)\Sigma_{i}^{-1}\right) \end{array}$$

$$\begin{array}{cc} \; & =\frac{4}{\lambda^{2}n}\psi_{\max}\mathrm{tr}\left(X_{i}\left(B_{i}^{*}-{\hat{B}}_{i}\right)\Sigma_{i}^{-2}{\left(B_{i}^{*}-{\hat{B}}_{i}\right)}^{⊤}X_{i}^{⊤}\right) \end{array}$$

$$\begin{array}{cc} \; & \leq \frac{4}{\lambda^{2}n}\frac{\psi_{\max}}{C_{1}-\lambda^{*}}\mathrm{tr}\left(X_{i}\left(B_{i}^{*}-{\hat{B}}_{i}\right)\Sigma_{i}^{-1}{\left(B_{i}^{*}-{\hat{B}}_{i}\right)}^{⊤}X_{i}^{⊤}\right). \end{array}$$

Since a common sparsity structure is shared across all platforms, the following is assumed:

$$\begin{array}{cc} M_{1}\left(\hat{B_{i}}\right) & \leq \frac{4\psi_{\max}}{\lambda^{2}n}\frac{1}{k(C_{1}-\lambda^{*})}\sum_{i=1}^{k}\mathrm{tr}\left[\left(X_{i}\left(B_{i}^{*}-{\hat{B}}_{i}\right)\right)\Sigma_{i}^{-1}{\left(X_{i}\left(B_{i}^{*}-{\hat{B}}_{i}\right)\right)}^{⊤}\right] \end{array}$$

(A21) $$\begin{array}{cc} \leq \frac{64\psi_{\max}}{\left(C_{1}-\lambda^{*}\right)}{\left(\frac{u^{1/2}}{\kappa_{1}}+\frac{s_{{\tilde{\rho }}^{*}}^{1/2}}{\kappa_{2}}\right)}^{2}. & \; \end{array}$$

This completes the proof of Theorem 1. □
